# Prognostic Value of HHLA2 in Patients with Solid Tumors: A Meta-Analysis

**DOI:** 10.3390/ijms25094760

**Published:** 2024-04-26

**Authors:** Agnieszka Kula, Miriam Dawidowicz, Sylwia Mielcarska, Elżbieta Świętochowska, Dariusz Waniczek

**Affiliations:** 1 Department of Oncological Surgery, Faculty of Medical Sciences in Zabrze, Medical University of Silesia, 41-808 Katowice, Poland; d201069@365.sum.edu.pl; 2 Department of Medical and Molecular Biology, Faculty of Medical Sciences in Zabrze, Medical University of Silesia, 41-800 Zabrze, Poland; d201109@365.sum.edu.pl (S.M.); eswietochowska@365.sum.edu.pl (E.Ś.)

**Keywords:** HHLA2, solid cancers, meta-analysis, overall survival (OS), progression-free survival (PFS), recurrence-free survival (RFS), disease-free survival (DFS)

## Abstract

HHLA2 is a checkpoint from the B7 family that can play a co-stimulatory or co-inhibitory role in cancer, depending on the binding receptor. The aim of this meta-analysis was to assess the relationship between HHLA2 levels and its impact on the prognosis of patients with solid cancers. The study used data from PubMed, Embase, Web of Science (WOS), Cochrane and SCOPUS databases. The R studio software was used for the data analysis. The study assessed overall survival (OS), disease-specific survival (DSS), progression-free survival (PFS), recurrence-free survival (RFS), and disease-free survival (DFS) by pooling appropriate hazard ratios (HR). Eighteen studies (2880 patients’ data) were included. High expression of HHLA2 was associated with worse OS (HR = 1.58, 95% CI: 1.23–2.03), shorter RFS (HR = 1.95, 95% CI: 1.38–2.77) and worse DFS (HR = 1.45, 95% CI: 1.01–2.09) in patients with solid cancers. The current study suggests that high expression of HHLA2 is associated with poorer prognosis in patients with solid cancers.

## 1. Introduction

Immune checkpoint blockade (ICB) therapies treat cancers by releasing the inhibiting signal and stimulating the host’s immune system. Immunotherapy blocking the PDL1/PD1 pathway has been a successful clinical advancement in the treatment of various cancers such as non-small cell lung cancer, melanoma, renal cell carcinoma, etc. [1]. Unfortunately, many tumors are resistant to current immunotherapy, and not every patient benefits from this innovative treatment [2]. Recently, much attention has been paid to studying immune pathways to find new targets for therapy. The search for new targets is intended to improve current immunotherapy and increase the number of treated patients. The human endogenous retrovirus-H Long repeat-associating 2 (HHLA2) is a member of the B7 family, and its gene was identified and first described in 1999 [3]. HHLA2 is an atypical B7 family checkpoint that differs from other members’ structures and lacks orthologs in mice or rats. This molecule is constitutively expressed in antigen-presenting cells (APC) in humans, in normal tissues, and is increased in various cancers such as breast, lung, thyroid, ovary, pancreas, liver, and cancers of gastrointestinal tracts. HHLA2 is characterized by a duality of functions, which determines the interaction with two receptors—co-stimulatory Transmembrane and immunoglobulin domain containing 2 (TMIGD2) and co-inhibitory Killer Cell Immunoglobulin Like Receptor, Three Ig Domains And Long Cytoplasmic Tail 3 (KIR3DL3) [4]. TMIGD2 is expressed in naïve T cells, memory T lymphocytes, tissue-resident T cells, NK cells, plasmacytoid dendritic cells, and innate lymphoid cells. The interaction between HHLA2 and TMIGD2 promotes the proliferation and differentiation of T cells and the activation of NK cells [5]. KIR3DL3 is expressed in CD8 and NK cells, and its relationship with HHLA2 inhibits T cells and mediates tumor resistance against NK cells. With the activation of T cells, the TMIGD2 receptor is gradually lost while the expression of KIR3DL3 increases, promoting the co-inhibitory abilities of HHLA2. Tumors may escape immune surveillance through the KIR3DL3-HHLA2 pathway; studying the mechanisms of this pathway may create a new target for immunotherapy [3].

The prognosis prediction value of HHLA2 for various cancers remains unclear. Several studies indicated unfavorable clinical outcomes associated with overexpression of HHLA2; however, various reports are identifying HHLA2 as a protective factor [6,7,8,9]. We conducted a meta-analysis to investigate the correlation between the expression of HHLA2 and clinical outcomes in patients with solid tumors. Our study elucidated the associations between HHLA2 expression and overall survival (OS), progression-free survival (PFS), disease-free survival (DFS), recurrence-free survival (RFS), and disease-specific survival (DSS). Our results highlight the prognostic value of HHLA2 expression in patients with solid tumors.

## 2. Results

### 2.1. Results of the Meta-Analysis

#### 2.1.1. Search Results

**Figure 1:**
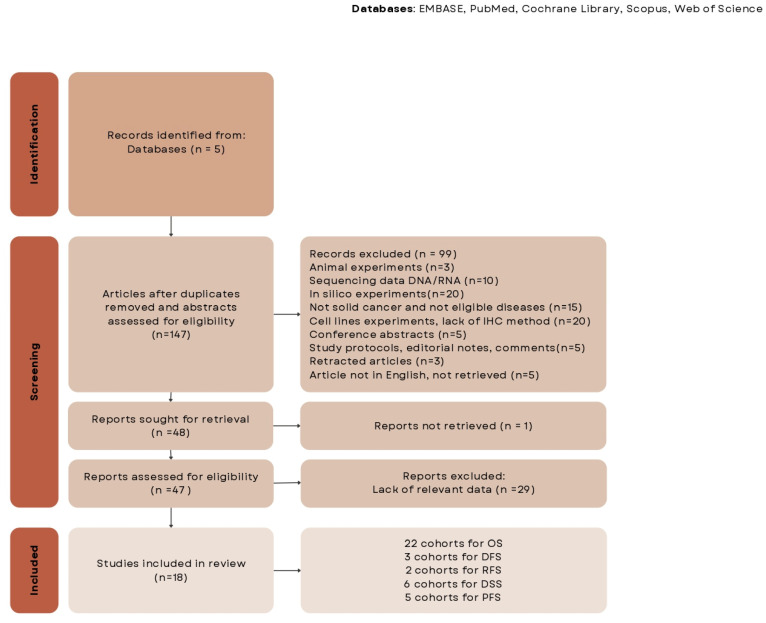
Study selection flowchart.

Figure 1 shows a flowchart of the study identification and selection procedure. In total, 18 studies involving 2880 patients with solid cancers met the inclusion criteria for analysis. All of them were retrospective cohort studies.

#### 2.1.2. HHLA2 and Survival Outcomes

**Figure 2:**
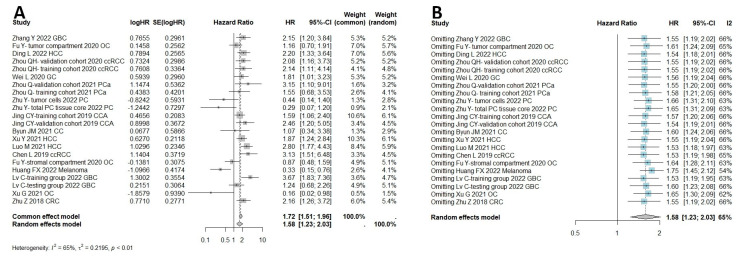
(**A**) Meta-analysis of overall survival with high HHLA2 expression in comparison to low HHLA2 expression in 2659 patients with solid tumors, as estimated by random effect model due to moderate heterogeneity among studies (I^2^ 65%). The summary HR shows unfavorable prognosis in the group with increased HHLA2 expression (HR 1.58, 95% CI: 1.23–2.03, *p* = 0.0003). Studies differed significantly in cut-off values for dichotomizing high and low expression (Supplementary Materials Figure S6). (**B**) One-leave meta-analysis for investigating the effects of removing single study from analysis on the association between HHLA2 expression and overall survival in solid tumors. Excluding a particular study from the meta-analysis did not result in significant effect on the overall survival.

**Figure 3:**
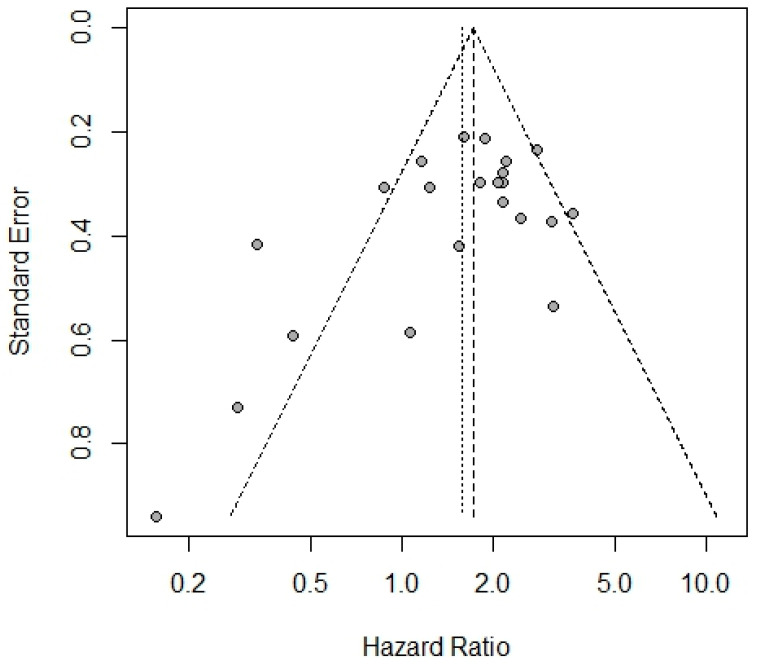
Funnel plots for evaluating potential publication bias on the association between HHLA2 high expression and overall survival in solid tumors. Egger’s test *p*-value = 0.039, Begg’s test *p*-value = 0.108.

**Figure 4:**
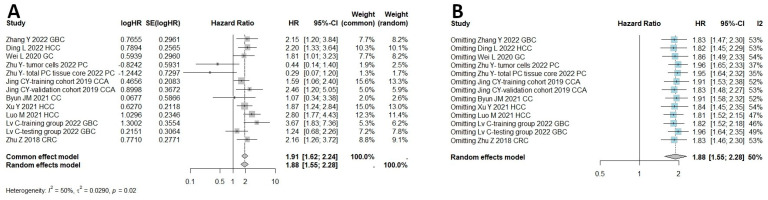
(**A**) Meta-analysis of overall survival with high HHLA2 expression in comparison to low HHLA2 expression in 1548 patients with gastrointestinal cancers as estimated by random effect model, due to moderate heterogeneity among studies (I^2^ 50%). The summary HR shows poor prognosis in the group with elevated HHLA2 expression (HR 1.88, 95% CI: 1.55–2.28, *p* < 0.0001). Similarly as in meta-analysis comparing OS in solid tumors, studies differed significantly in cut-off values for dichotomizing high and low expression. (**B**) One-leave meta-analysis for investigating the effects of removing single study from analysis on the association between HHLA2 expression and overall survival in gastrointestinal cancers. Excluding a particular study from the meta-analysis did not result in significant effect on the overall survival.

In total, 18 studies were qualified to assess the connection between HHLA2 and OS, including both univariate and multivariate analysis. If univariate and multivariate HRs existed, the latter was selected to minimize the bias. Separate multivariate and univariate analyses are included in Supplementary Materials (Figures S1 and S2). The results of the meta-analysis showed that high expression of HHLA2 was associated with shorter OS (random effect model HR = 1.58, 95% CI: 1.23–2.03). Heterogeneity was I^2^ = 65% and *p* < 0.01 (Figure 2A). Figure 2B shows a one-leave meta-analysis for investigating the effects of removing a single study from analysis on the association between HHLA2 expression and overall survival in solid tumors. Excluding a particular study from the meta-analysis did not result in significant effect on the overall survival. Figure 3 shows the funnel plots for evaluating potential publication bias regarding the association between HHLA2 high expression and overall survival in solid tumors (Egger’s test *p*-value = 0.039, Begg’s test *p*-value = 0.108). We analyzed a subset of gastrointestinal cancers in which a shorter overall survival time was observed in patients with high HHLA2 expression OS (random effect model, HR = 1.88, 95% CI: [1.55, 2.28]), as shown in Figure 4A. We performed a one-leave meta-analysis to investigate the effects of removing a single study from the analysis on the association between HHLA2 expression and overall survival in gastrointestinal cancers (Figure 4B). 

**Figure 5:**
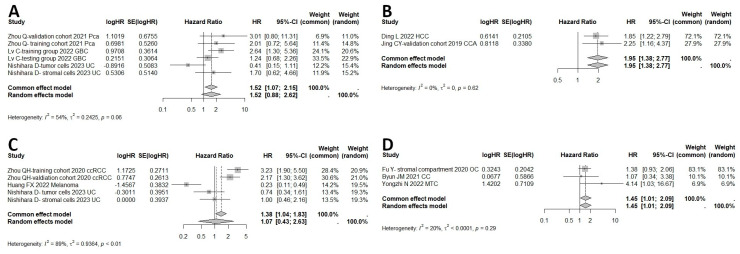
(**A**) Meta-analysis of disease-specific survival (DSS) with high HHLA2 expression in comparison to low HHLA2 expression in 607 patients with solid tumors as estimated by random effect model, due to moderate heterogeneity among studies (I^2^ 54%). The summary HR is not significant (*p* = 0.88). (**B**) Meta-analysis of recurrence-free survival (RFS) with high HHLA2 expression in comparison to low HHLA2 expression in 254 patients with solid tumors (only two studies included HCC and CCA reported RFS) as estimated by common effect model. The HR shows unfavorable prognosis in the group with elevated HHLA2 expression (HR 1.95, 95% CI: 1.38–2.77, *p* = 0.0002). (**C**) Meta-analysis of progression-free survival (PFS) with high HHLA2 expression in comparison to low HHLA2 expression in 654 patients with tumors as estimated by random effect model. Especially important to note is high heterogeneity among studies (I^2^ 89%). The pooled HR is not significant (*p* = 0.88). (**D**) Meta-analysis of disease-free survival (DFS) with high HHLA2 expression in comparison to low HHLA2 expression in 246 patients with solid tumors as estimated by common effect model due to low heterogeneity among studies (I^2^ 20%). Among analyzed studies, only three reported DFS (included ovarian cancer, colorectal cancer, and medullary thyroid cancer). The summary HR shows worse prognosis in the group with high HHLA2 expression, but the result was at the limit of statistical significance (HR 1.45, 95% CI: 1.01–2.09, *p* = 0.0448).

The results among all cancers indicated that high expression of HHLA2 in human tumor tissue was associated with shorter RFS compared to low expression of HHLA2 (HR = 1.95, 95% CI: 1.38–2.77) as shown in Figure 5B. The meta-analysis of the HR rate for DFS rate showed that the patients with high HHLA2 expression had worse DFS than those with low expression (common effect model, HR = 1.45, 95% CI: 1.01–2.09) (Figure 5D). 

**Figure 6:**
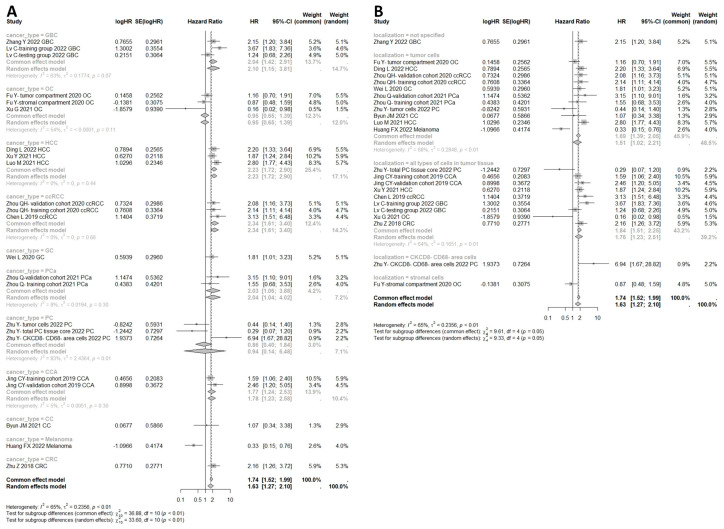
(**A**) Subgroup analysis for the relationship between HHLA2 and overall survival. Grouped by different cancers. High expression of HHLA2 was associated with poor OS in gallbladder cancer (HR = 2.10, 95% CI: 1.15–3.81), hepatocellular carcinoma (HR = 2.23, 95% CI: 1.72–2.90), clear cell renal cell carcinoma (HR = 2.34, 95% CI: 1.61–3.40), prostate carcinoma (HR = 2.03, 95% CI: 1.06–3.88), cholangiocarcinoma (HR = 1.77, 95% CI: 1.24–2.53), apart from pancreatic cancer (HR = 0.94, 95% CI: 0.14–6.48) and ovarian cancer (HR = 0.95, 95% CI: 0.65–1.39). (**B**) Subgroup analysis for the relationship between HHLA2 and overall survival. Grouped by localization of HHLA2 expression. High expression of HHLA2 was associated with poor OS in every localization: tumor cells (HR = 1.51, 95% CI: 1.02–2.21), stromal cells (HR = 1.63, 95% CI: 1.27–2.10), all types of cells in tumor tissues (HR = 1.76, 95% CI: 1.23–2.51).

The meta-analysis of the HR rate for DSS for the prostate, gallbladder, and urothelial cancer is shown in Figure 5A. There was no significance between the high expression of HHLA2 and DSS (Random effect model, HR = 1.52, 95% CI: 0.88–2.62). Additionally, there was no association between high HHLA2 expression and shorter PFS (random effect model HR = 1.07, 95% CI: 0.43–2.63) (Figure 5C). Multivariate subgroup analysis by cancer type revealed that high expression of HHLA2 was associated with poor OS in gallbladder cancer (HR = 2.10, 95% CI: 1.15–3.81), hepatocellular carcinoma (HR = 2.23, 95% CI: 1.72–2.90), clear cell renal cell carcinoma (HR = 2.34, 95% CI: 1.61–3.40), prostate carcinoma (HR = 2.03, 95% CI: 1.06–3.88), and cholangiocarcinoma (HR = 1.77, 95% CI: 1.24–2.53), but not in pancreatic cancer (HR = 0.94, 95% CI: 0.14–6.48) or ovarian cancer (HR = 0.95, 95% CI: 0.65–1.39), as shown in Figure 6A. We performed a subgroup analysis of the relationship between HHLA2 and overall survival, grouped by localization of HHLA2 expression. The subgroup analysis revealed that high expression of HHLA2 was associated with poor OS in every localization: tumor cells (HR = 1.51, 95% CI: 1.02–2.21), stromal cells (HR = 1.63, 95% CI: 1.27–2.10) and all types of cells in tumor tissues (HR = 1.76, 95% CI: 1.23–2.51), as shown in Figure 6B. The supplementary materials include the following figures: forest plot of studies evaluating HRs of high HHLA2 expression and OS univariate in solid tumors (Figure S1A), funnel plots for evaluating potential publication bias on the association between HHLA2 high expression and overall survival in solid tumors. OS univariate (Figure S1B), one-leave meta-analysis for investigating the effects of particular studies on the association between HHLA2 expression and overall survival in solid tumors. OS univariate (Figure S1C), forest plot of studies evaluating HRs of high HHLA2 expression and OS in solid tumors. OS multivariate analysis. (Figure S2A), funnel plots for evaluating potential publication bias on the association between HHLA2 high expression and overall survival in solid tumors. OS multivariate analysis (Figure S2B), one-leave meta-analysis for investigating the effects of particular studies on the association between HHLA2 expression and overall survival in solid tumors. OS multivariate (Figure S2C), subgroup analysis for the relationship between HHLA2 and overall survival. Grouped by different cancers. Univariate analysis (Figure S3A), forest plot of studies evaluating HRs of high HHLA2 expression and OS in gastrointestinal cancers. Univariate analysis (Figure S3B), one-leave meta-analysis investigating the effects of excluding particular studies from analysis on the relationship between high HHLA2 expression and OS in gastrointestinal cancers. Univariate analysis (Figure S3C), subgroup analysis for the relationship between HHLA2 and overall survival. Grouped by different cancers. Multivariate analysis (Figure S4A), forest plot of studies evaluating HRs of high HHLA2 expression and OS in gastrointestinal cancers.Multivariate analysis (Figure S4B), funnel plots for evaluating potential publication bias on the association between HHLA2 high expression and overall survival in gastrointestinal cancers. Multivariate analysis (Figure S4C), forest plot for DFS. Univariate analysis (Figure S5A), forest plot for DFS. Multivariate analysis (Figure S5B), forest plot for PFS. Univariate analysis (Figure S5C), forest plot for PFS. Multivariate analysis (Figure S5D), forest plot for DSS. Univariate analysis (Figure S5E), forest plot for DSS. Multivariate analysis (Figure S5F), subgroup analysis for the association between HHLA2 and overall survival grouped by cut off of HHLA2 low/high expression (Figure S6). 

## 3. Discussion

Immunotherapy represents a promising and rapidly advancing treatment modality; however, it has many limitations. Some cancers demonstrate resistance to available immunotherapies, and many patients do not benefit from these methods [2,6,9]. Another critical issue is the need for a useful predictive marker that indicates sensitivity or resistance to a specific type of therapy and precisely determines the patient’s prognosis. The search for new checkpoints that will be appropriate targets for blockade and prognostic markers is crucial for developing immunotherapy. HHLA2 is a relatively new checkpoint from the B7 family that plays a vital role in immune escape and has been reported as a factor with prognostic significance in many studies. Still, the results were often controversial and needed clarification [8,10].

HHLA2 is involved in several signaling pathways in tumorigenesis, such as cell cycle regulation, apoptosis, proliferation, and epithelial-mesenchymal transition. Silencing HHLA2 could be helpful in cancer treatment because it has been shown to improve clinicopathological conditions such as survival and decrease tumor size, cancer cell invasion, migration, and proliferation. However, the prognosis prediction value of HHLA2 for various cancers is still unclear.

Although certain studies suggest that overexpression of HHLA2 may lead to unfavorable clinical outcomes, there are multiple reports that highlight the protective properties of HHLA2.

We conducted a systematic review and meta-analysis of the available studies. Our meta-analysis included 18 studies, and the study group consisted of 2880 patients with solid tumors. The results of the meta-analysis showed that high expression of HHLA2 was associated with poor prognosis. High expression of HHLA2 was a risk factor for OS (HR = 1.58, 95% CI: 1.23–2.03) and RFS (HR = 1.95, 95% CI: 1.38–2.77). Additionally, the meta-analysis of the HR rate for DFS rate showed that patients with high HHLA2 expression had worse DFS than those with low expression. We analyzed a subset of gastrointestinal cancers in which a shorter overall survival time was observed in the patients with high HHLA2 expression OS (random effect model, HR = 1.88, 95% CI: [1.55, 2.28]). The meta-analysis results comparing PFS showed no association between high HHLA2 expression and shorter PFS (HR = 1.07, 95% CI: 0.43–2.63), likewise between HHLA2 and DSS (HR = 1.52, 95% CI: 0.88–2.62). Moreover, the analysis revealed that HHLA2′s high expression was associated with poor OS regardless of its location within the tumor. Our meta-analysis findings on the correlation between HHLA2 and overall survival align with the research conducted by Zhang et al. in 2021, which focused on the Chinese population. Zhang et al. found that high HHLA2 expression was significantly associated with shorter OS. They also discovered that in the subgroup analysis by cancer type, HHLA2 overexpression correlated with poor OS in patients with clear cell renal cell carcinoma, gastric cancer, cholangiocarcinoma, lung cancer, and other cancer types, but not in patients with ovarian cancer [11]. Our study, which involved a much more extensive research group, yielded similar results. Several studies have demonstrated that overexpression of HHLA2 in tumor cells is linked to adverse clinical outcomes and reduced survival rates in patients with various types of cancer, such as prostate cancer [12], hepatocellular carcinoma [13], lung adenocarcinoma [10], gastric cancer [14], bladder urothelial carcinoma [15], cholangiocarcinoma [16], colorectal carcinoma [17], osteosarcoma [18], and breast cancer [19]. Our meta-analysis findings are consistent with the existing research on the correlation between HHLA2 and clinical outcomes. Our comprehensive analysis of studies conducted on solid tumors has led us to postulate that HHLA2 may serve as an unfavorable prognostic marker. However, despite the weight of the evidence, some studies have yielded contradictory results, which require further investigation and analysis. Guocai Xu et al. reported that HHLA2 expression is an independent prognostic factor that predicted improved survival in ovarian cancer; moreover, they found that overexpressing HHLA2 inhibited the proliferation of ovarian cancer cells [20]. In unresectable and advanced melanoma, Huang FX et al. found that HHLA2 has important values in predicting the response to ICB and indicating improved PFS and OS [21]. Such variable results may come from the dual effect of HHLA2 on the immune system. HHLA2 binds to the TMIGD2 receptor on naive T cells and plays a co-stimulatory role in the immune system. The interaction of HHLA2 and TMIGD2 promotes T cell proliferation, T cell differentiation, and NK cell activation. On the other hand, HHLA2 binds to the KIR3DL3 receptor, a robust immune system inhibitor. The relation of HHLA2 to KIR3Dl3 is essential for NK cell and CD8+ T cell suppression. With the activation of T cells, the TMIGD2 receptor is gradually lost while the expression of KIR3DL3 increases, promoting the co-inhibitory abilities of HHLA2. The evolving landscape of HHLA2 research deepens our understanding of its immunomodulatory functions and offers new opportunities for employing its therapeutic potential in cancer treatment. Recently, ideas have been developed to block the KIR3DL3 receptor while maintaining the positive effect of TMIGD2. Bhatt et al. first generated monoclonal antibodies against the HHLA2/KIR3DL3 pathway that blocked the KIR3DL3 inhibitory activity while keeping the TMIGD2 immune-stimulatory effects of HHLA2 [22]. This discovery opened avenues for exploring the intricate network of immune checkpoint pathways and their interplay in regulating immune responses within the tumor microenvironment. Furthermore, the interaction between HHLA2 and its receptors has been implicated in regulating immune cell functions beyond T cells, including the modulation of dendritic cell activity and cytokine production, suggesting broader implications for immune regulation in cancer. A Phase I clinical trial began in July 2023, using a monoclonal antibody called NPX887. The antibody targets KIRD3LD3 to reactivate the immune cells that have become exhausted. This treatment is intended for recurrent or metastatic solid tumors, such as non-small cell lung carcinoma (NSCLC), renal cell carcinoma (RCC), colorectal carcinoma (CRC), cholangiocarcinoma (CCA), pancreatic cancer (PDAC), urothelial carcinoma (UCC), gastric/gastroesophageal carcinoma, triple-negative breast carcinoma, endometrial carcinoma, cervical cancer, osteosarcoma, and prostate cancer. https://classic.clinicaltrials.gov/ct2/show/NCT06240728 (accessed on 1 February 2024).

The results of this meta-analysis showed significant heterogeneity between studies. The most important cause of high heterogeneity could be different cut-off values of HHA2 expression to dichotomize high and low levels in cohorts. The majority of studies used H-scores to assess the level of HHLA2 expression, which were multiplications of the percentage expression in the tumors and the intensity of the staining; their cut offs were often calculated using dedicated software such as X-tile (New Haven, CT, USA, version 3.6.1) [13,23]. These methods that divide tumors into high and low expression groups make the studies uncomparable and confuse the meta-analysis results. Additionally, it was reported in previous studies that HHLA2 may exhibit both protumor and antitumor activity depending on tumor type, which suggests a more complicated association between HHLA2 expression and prognosis in different cancers. Sensitivity analysis and publication bias proved that the results of OS analysis were strong and reliable. On the contrary, sensitivity analyses of meta-analyses comparing DFS and PFS revealed a lack of reliability and indicated the contrary influence of each study on the pooled effect size. It must be highlighted that an extremely limited number of studies reported DFS (k = 3).

Several limitations should be noted in our study. First, HHLA2 is a relatively recently discovered molecule, so the amount of research is limited. Secondly, the meta-analysis included studies only from Asian populations; due to differences in genetics among populations, studies should be also conducted on other racial groups. Indeed, the results will become more accurate as research on HHLA2 increases and occurs in more diverse populations. We plan to extend our previous study on HHLA2 expression in colorectal cancer of the European population with a 5-year follow-up. Third, there were limited data to assume differences in the correlations between HHLA2 levels and treatment effects across most tumors. Moreover, the trials used different cut-off values to dichotomize HHLA2 expression levels, complicating the meta-analytic studies. Finally, multivariate analyses may be more optimal for estimating the effect size than univariate analyses, which have only been reported in a few studies. In our analysis, if univariate and multivariate HRs existed, the latter was selected. Given this study’s limitations, it is imperative to conduct further, more rigorous research in diverse populations to ascertain the relationship between HHLA2 expression and survival outcomes. This will help to fill the gaps in our current understanding and pave the way for more comprehensive and detailed studies.

## 4. Materials and Methods

### 4.1. Meta-Analysis

#### 4.1.1. Literature Search Criteria and Outcomes

The following systematic review and meta-analysis adhered to the Preferred Reporting Items for Systematic Reviews and Meta-Analysis (PRISMA) guidelines. This review was registered on the PROSPERO platform (CRD42023416723). Databases (PubMed, EMBASE, Cochrane, Web of Science, SCOPUS) were systematically searched to identify articles evaluating the prognostic value of HHLA2 expression in solid tumors published before September 2023. Articles were identified with search terms including “HHLA2” AND “cancer” AND “carcinoma” AND “malignancy” AND “tumor”. Outcomes included overall survival (OS), disease-free survival (DFS), recurrence-free survival (RFS), progression-free survival (PFS), and disease-specified survival (DSS). The language of the restricted search was English.

#### 4.1.2. Inclusion and Exclusion Criteria

Inclusion criteria included patients diagnosed with solid cancer before enrollment, randomized controlled trials (RCTs) or observational studies, assessment of HHLA2 expression by IHC method, clinical outcome presented as hazard ratio for meta-analysis, or presence of Kaplan–Meier curve with a number at-risk table.

Exclusion criteria included non-solid and nervous system cancer, case reports, single-cell sequencing data, animal experiments, meta-analyses, network meta-analyses, conference presentations, or study protocols.

#### 4.1.3. Study Selection and Data Extraction

The review was performed by two independent reviewers (AK and MD). A third independent author (SM) was consulted in case of discrepancies. The following information was extracted from the articles: basic information, cancer type, study design features, clinical, and pathological characteristics of the patients, methodology details, clinical outcomes details, details about HHLA2, including expression location and cut-off value determining high expression, HR estimation method (univariate and multivariate analysis), and HR ratio. If the study did not report HR ratio, but survival curve with the number at-risk table was published, the HR values were reconstructed using WebPlotDigitizer v4.7 and algorithm developed by Guyot P in R Studio [24].

#### 4.1.4. Study Characteristics

The characteristics of the included studies are shown in Table 1. Studies were published between 2018 and 2023. Ding L [9], Xu Y [7], and Luo M [13] reported survival outcomes of hepatocellular carcinoma (HCC). The validation and training cohort was examined for renal cell carcinoma (RCC) by Zhou QH [12]; we also used RCC results from Chen L’s [8] research. Zhang Y [23] and Lv C [25] with training and testing cohort reported outcomes of gallbladder cancer (GBC), Wei L [14] reported gastric cancer (*GC*), Zhou Q [12] reported results from validation and training cohort of prostate carcinoma (PCa), Zhu Y [26] reported results from tumor cells and tissue core groups of pancreatic cancer (PC), Byun JM [27] reported cervical cancer (CvC), Fu Y [28] and Xu G [7,20] reported ovarian cancer (OC), Huang FX [21] reported malignant melanoma (MM), Niu Y [29] reported medullary thyroid cancer (MTC), Nishihara D [15] reported urothelial cancer (UC) for tumur and stromal cells, Zhu Z [17] reported colorectal cancer (CRC), and Jing CY [16] reported survival outcomes of patients with cholangiocarcinoma (CCA). In total, 18 studies were qualified to assess the connection between HHLA2 and OS, including both univariate and multivariate analysis; if both univariate and multivariate HRs existed, the latter was selected to minimize bias. We extracted Ding L, Wei L, Zhu Y, Jing CY, Xu Y, Luo M, LV C, Zhu Z’s results from all articles to perform analysis for gastrointestinal cancers.

#### 4.1.5. Strategy for Meta-Analysis

All statistical analyses in this study were performed with R software (version 4.0.3). In terms of methods for estimating HR, multivariate analysis was used; from the remaining articles which did not report multivariate analysis, the univariate models were used. Statistical heterogeneity was estimated by chi-square Cochran’s Q-test and Higgins I^2^ statistics. I^2^ values were interpreted as follows: 25–50%—low heterogeneity, 50–75%—moderate heterogeneity, above 75%—high heterogeneity, according to J. P. Higgins and Thompson, 2002 [30]. I^2^ < 50% and *p* value > 0.05 indicated no substantial heterogeneity. Then, a fixed-effects model was used to pool the value of HR and 95% confidence interval. The random-effects model was applied to determine the reasons for heterogeneity, subgroup and sensitivity analysis were performed. Sensitivity analysis was used to test the effect of the exclusion one study each time. The publication bias assessment was performed by funnel plot, Begg’s and Egger’s tests *p* < 0.05 was considered significant. All statistical analyses were performed using R software (version 4.0.3).

#### 4.1.6. Quality Assessment

Two reviewers (SM, MD) independently assessed the quality of eligible studies independently by using the Newcastle–Ottawa Quality Assessment Scale (NOS). The NOS assessed the quality of the studies from the aspects of selection, comparability, and exposure, with a total score ranging from 0 to 9 points. Due to the fact that overall survival, as the most frequently reported prognostic parameter, is not sufficient to be an indicator of study quality, one star was given if the study reported only OS, and two stars if DFS, DSS or RFS were also presented. The NOS points in the category of ‘Comparability of cohorts on the basis of the design or analysis were given as follows: one star to the study reporting hazard ratio and two stars if HR was obtained from multivariate analysis [19]. More than six points was defined as high-quality (Table 2).

## 5. Conclusions and Future Work

Our work highlights the prognostic significance of HHLA2 expression in patients with solid tumors. HHLA2 serves as a valuable predictor of survival prognosis in patients with solid cancers. Our results indicated that this checkpoint correlated with shorter overall survival, worse disease-free survival, and recurrence-free survival. Further research into the pathways in which HHLA2 is involved may contribute to finding a new potential target for immunotherapy and a predictive factor. Among the diverse immunotherapies, immune checkpoint inhibitors stand out as hopeful, poised to revolutionize the future of cancer treatment. The unique dualistic interaction of HHLA2 with its two receptors, a co-stimulatory receptor, TMIGD2, and a co-inhibitory, KIR3DL3, holds immense potential to unblock the immune system and stimulate it, thereby amplifying the therapeutic effect. Taking into account the positive function of the TMIGD2 receptor, a desirable phenomenon in therapy would be to block the interaction of HHLA2 with KIR3DL3 only. Targeting the HHLA2/KIR3DL3/TMIGD2 pathway, which we intend to explore in the future work, may be beneficial for cancer treatment and improve clinicopathological conditions such as survival. In the prospect of the potential immunotherapies targeting HHLA2, it is essential to establish its co-expression patterns with other immune checkpoint genes in various types of cancer, as they could influence the effects of such treatment. While our research has illuminated the prognostic value of HHLA2 expression in patients with solid tumors, further exploration is clearly needed to fully understand its mechanisms. 

## Figures and Tables

**Table 1:** Basic characteristics.

Author	Year	Patient Source	Sample Size	Method	Cancer Type	HHLA2 + Expression	Outcome	HR Ratio	Multivare (M)/ Univare (U)	Cohort	Cell Types	Cut Off	Citation
Ding L	2022	China	189	IHC	HCC	0.481	OS/RFS	Reported	U/M		Tumor cells	H-score ≥ 5	[9]
Zhou QH	2020	China	197	IHC	ccRCC	0.411	OS/PFS	Reported	U/M	Validation cohort	Tumor cells	>20%	[6]
Zhou QH	2020	China	206	IHC	ccRCC	0.44	OS/PFS	Reported	U/M	Training cohort	Tumor cells	>20%	[6]
Zhang Y	2022	China	89	IHC	GBC	0.50	OS	Reported	U		Not specified	H-score > 80	[23]
Wei L	2020	China	182	IHC	GC	0.68	OS	Reported	U/M		Tumor cells	final score ≥ 8	[14]
Zhou Q	2021	China	113	IHC	PCa	0.68	OS/DSS	Reported	U/M	Validation cohort	Tumor cells	H score > 80	[12]
Zhou Q	2021	China	126	IHC	PCa	0.69	OS/DSS	Reported	U/M	Training cohort	Tumor cells	H score > 80	[12]
Zhu Y	2022	China	63	IHC	PC	0.81	OS	Reported	U/M	Tumor cells cohort	Tumor cells	H score > 80	[26]
Zhu Y	2022	China	63	IHC	PC	0.67	OS	Reported	U/M	Tissue core cohort	All types of cells	H score > 80	[26]
Byun J M	2021	Korea	76	IHC	CvC	0.81	OS/DFS	Reported	U/M		Tumor cells	staining 0, +/++, +++	[27]
Xu Y	2021	China	205	IHC	HCC	0.33	OS	Reported	U/M		All types of cells	NA	[7]
Luo M	2021	China	202	IHC	HCC	0.51	OS	Reported	U/M		Tumor cells	IRS > 3	[13]
Chen L	2019	China	86	IHC	RCC	0.30	OS	Reported	U/M		All types of cells	H score > 90	[8]
Fu YY	2020	China	119	IHC	OC	0.54	OS/DFS	Reported	U/M	Stromal compartment	Stromal cells	>6.13%	[28]
Fu YY	2020	China	119	IHC	OC	0.50	OS/DFS	Reported	U/M	Tumor compartment	Tumor cells	>31.51%	[28]
Huang FX	2022	China	81	IHC	MM	0.22	OS/PFS	Reported	U/M		Tumor cells	>50%	[21]
Lv C	2022	China	95	IHC	GBC	0.53	OS/DSS	Reported	U/M	Validation cohort	All types of cells	H score > 91	[25]
Lv C	2022	China	103	IHC	GBC	0.60	OS/DSS	Reported	U/M	Training cohort	All types of cells	H score > 90	[25]
Niu Y	2022	China	51	IHC	MTC	0.31	DFS	Reported	M		Tumor cells	>50%	[29]
Xu G	2021	China	64	IHC	OC	0.17	OS	Reported	U/M		All types of cells	>0%	[20]
Nishihara D	2023	Japan	85	IHC	UC	0.68	DSS/PFS	Reported	U/M	Tumor cells cohort	Tumor cells	>20%	[15]
Nishihara D	2023	Japan	85	IHC	UC	0.55	DSS/PFS	Reported	U/M	Tissue core cohort	Stromal cells	>20%	[15]
Zhu Z	2018	China	63	IHC	CRC	0.47	OS	Reported	U/M		All types of cells	H score > median	[17]
Jing CY	2019	China	153	IHC	CCA	0.49	OS	Reported	M	Training cohort	Tumor cells	NA	[16]
Jing CY	2019	China	65	IHC	CCA	0.68	OS/RFS	Reported	M	Validation cohort	Tumor cells	NA	[16]

Immunohistochemistry (IHC), overall survival (OS), progression-free survival (PFS), disease-free survival (DFS), disease-specific survival (DSS), recurrence-free survival (RFS), hepatocellular carcinoma (HCC), renal cell carcinoma (RCC), gallbladder cancer (GBC), gastric cancer (GC), prostate carcinoma (PCa), pancreatic cancer (PC), cervical cancer (CvC), ovarian cancer (OC), malignant melanoma (MM), medullary thyroid cancer (MTC), urothelial cancer (UC), colorectal cancer (CRC), cholangiocarcinoma (CCA), not available (NA)

**Table 2:** Newcastle–Ottawa Scale for assessing the quality of studies in meta-analysis.

Study	Selection				Comparability	Exposure			Scores	Citation
	Representativeness of the Exposed Cohort	Selection of the Nonexposed Cohort	Ascertainment of Exposure	Demonstration That Outcome of Interest Was Not Present at Start of Study	Comparability of Cohorts on the Basis of the Design or Analysis	Assessment of Outcome	Was Followed-Up for Long Enough for Outcomes to Occur	Adequacy of Follow Up of Cohorts		
Ding L, 2022	★	★	★	-	★★	★	-	★	7	[9]
Zhou QH, 2020	★	★	★	-	★★	★	★	★	8	[6]
Zhang, 2022	★	★	★	-	★	-	★	★	6	[23]
Wei L, 2020	★	★	★	-	★★	-	★	★	7	[14]
Zhou Q, 2021	★	★	★	-	★★	★	★	★	8	[12]
Zhu Y, 2022	★	★	★	-	★★	-	★	-		[26]
Byun J M, 2021	★	★	★	-	★★	★	★	★	8	[27]
Xu Y, 2021	★	★	★	-	★★	-	★	★	7	[7]
Luo M, 2021	★	★	★	-	★★	-	★	★	7	[13]
Chen L, 2019	★	★	★	-	★★	-	★	★	7	[8]
Fu YY, 2020	★	★	★	-	★★	★	★	★	8	[28]
Huang FX, 2022	★	★	★	-	★★	★	-	★	7	[21]
Chao LV, 2022	★	★	★	-	★★	★	★	★	8	[25]
Niu Y, 2022	★	★	★	-	★★	★	★	★	8	[29]
Xu G, 2021	★	★	★	-	★★	-	★	★	7	[20]
Nishihara D, 2023	★	★	★	-	★★	★	★	★	8	[15]
Zhu Z, 2018	★	★	★	-	★★	-	★	★	7	[17]
Jing CY, 2019	★	★	★	-	★★	★	★	★	8	[16]

Identify ‘high’ quality choices with a ‘star’. A maximum of one ‘star’ for each item within the ‘Selection’ and ‘Exposure’ categories; maximum of two ‘stars’ for ‘Comparability’. When the ‘stars’ add up to ≥6 for a single piece of literature, the included literature is considered to be of high quality.

## Data Availability

The original contributions presented in the study are included in the article/supplementary materials, further inquiries can be directed to the corresponding author/s.

## Supplementary Materials

The supporting information can be downloaded at: https://www.mdpi.com/article/10.3390/ijms25094760/s1.
