# Supplementary material for: Prognostic Value of HHLA2 in Patients with Solid Tumors: A Meta-Analysis
Source: Int J Mol Sci. 2024 Apr 26;25(9):4760. doi: 10.3390/ijms25094760 (PMC11083681; doi:10.3390/ijms25094760)
Supplement: Supplementary file 1 [file ijms-25-04760-s001.zip › ijms-2957053-supplementary.pdf]

Supplementary Materials

Figure S1A

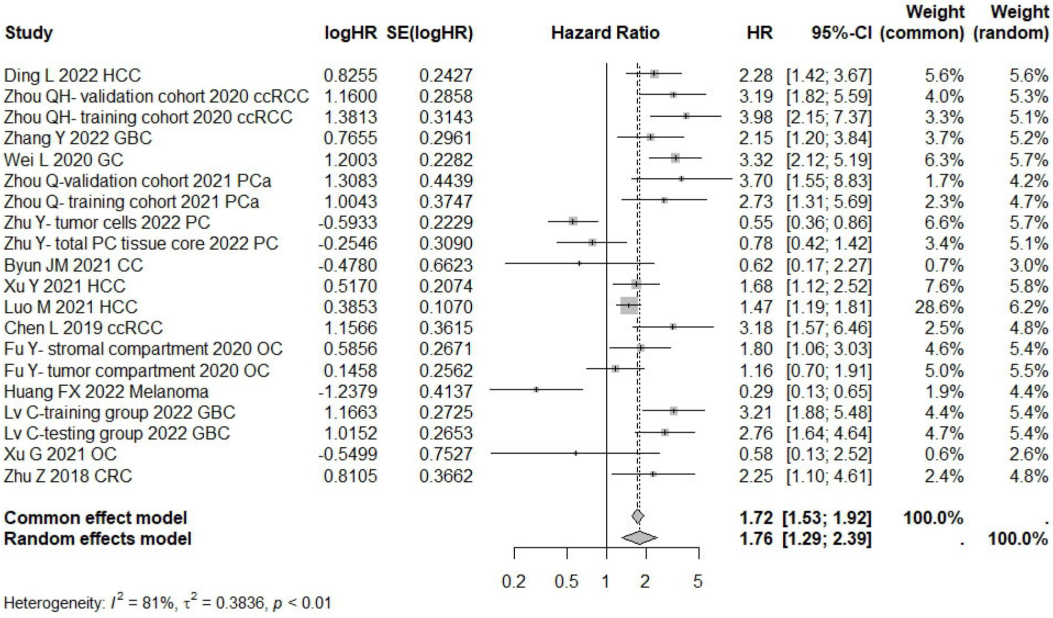

Figure S1A Forest plot of studies evaluating HRs of high HHLA2 expression and OS univariate in solid tumors. High expression of HHLA2 was associated with shorter OS (HR=1.76 95% CI: 1.29-2.39).

Figure S1B

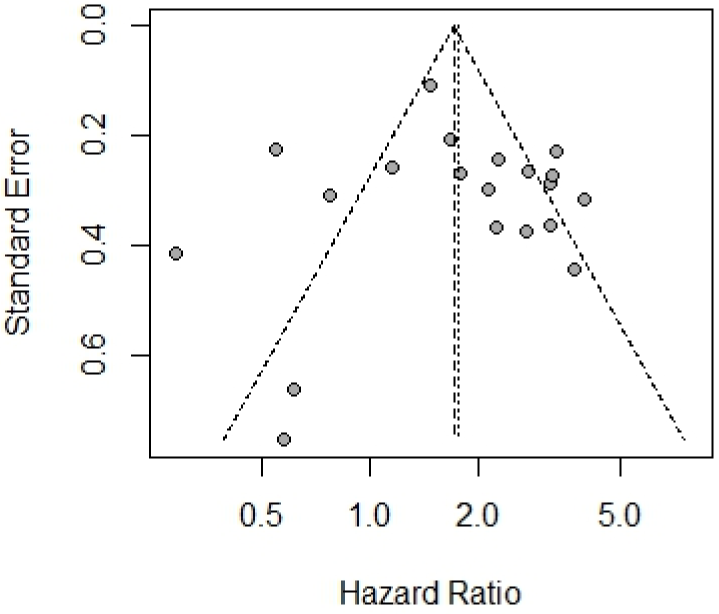

Figure S1B Funnel plots for evaluating potential publication bias on the association between HHLA2 high expression and overall survival in solid tumors. OS univariate.

Figure S1C

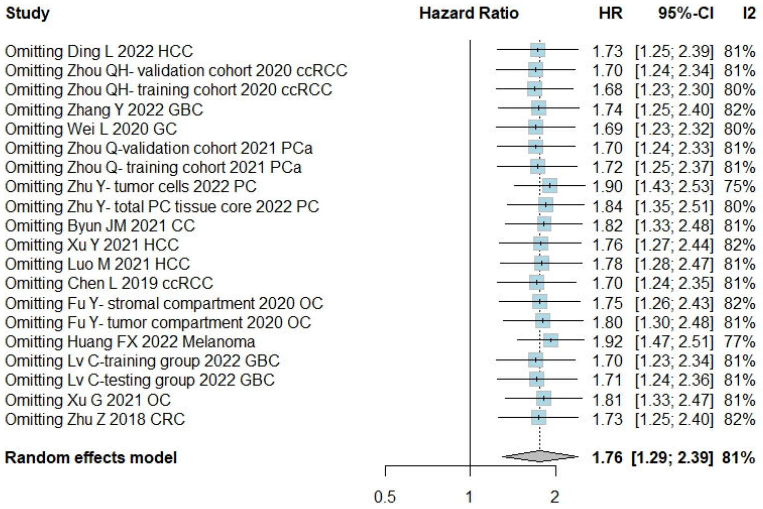

Figure S1C One-leave metaanalysis for investigating the effects of particular studies on the association between HHLA2 expression and overall survival in solid tumors. OS univariate.

Figure S2A

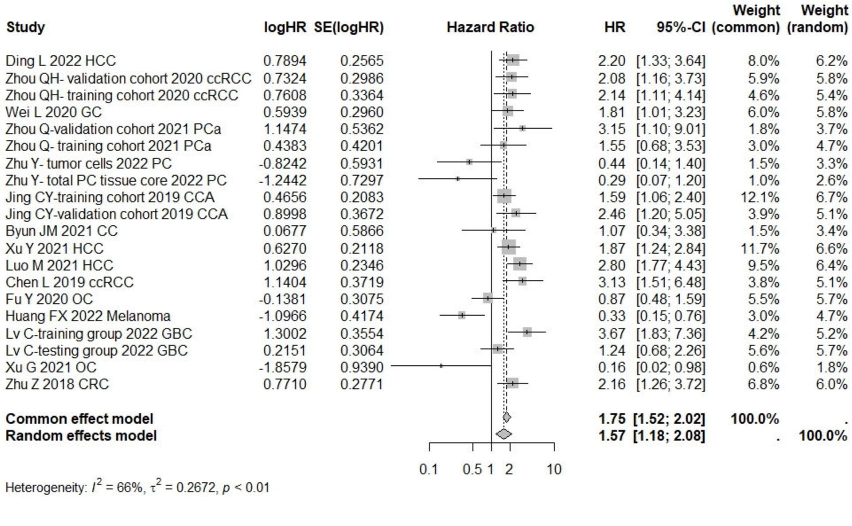

Figure S2A Forest plot of studies evaluating HRs of high HHLA2 expression and OS in solid tumors. OS multivariate analysis. High expression of HHLA2 was associated with shorter OS (HR=1.57 95% CI: 1.18-2.08).

Figure S2B

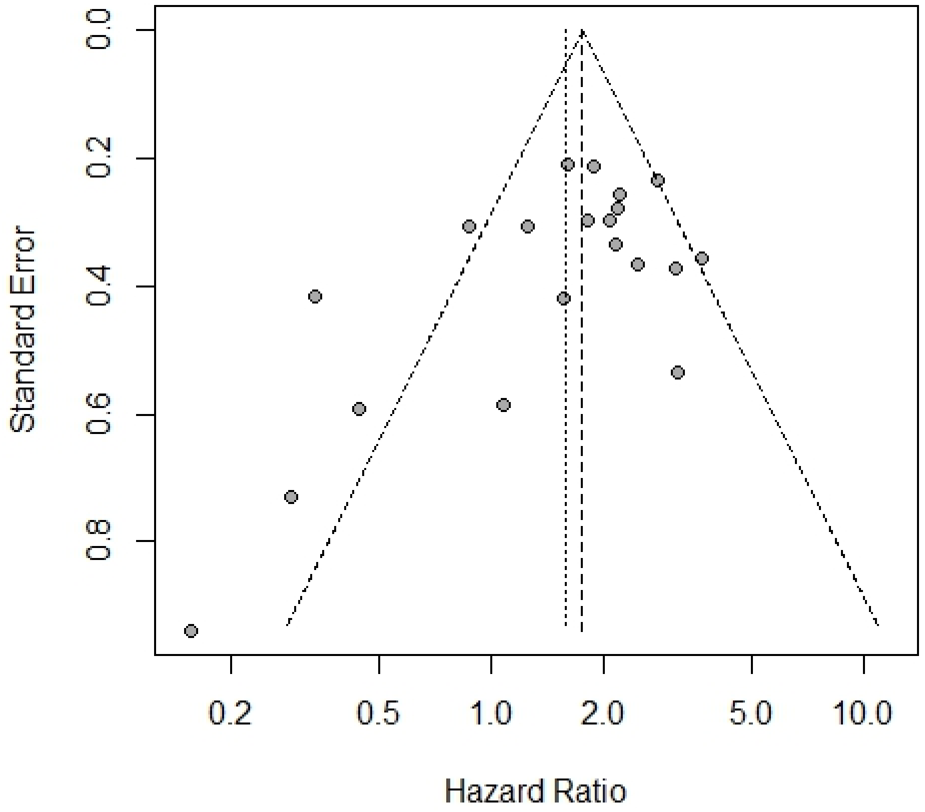

Figure S2B Funnel plots for evaluating potential publication bias on the association between HHLA2 high expression and overall survival in solid tumors. OS multivariate analysis.

Figure S2C

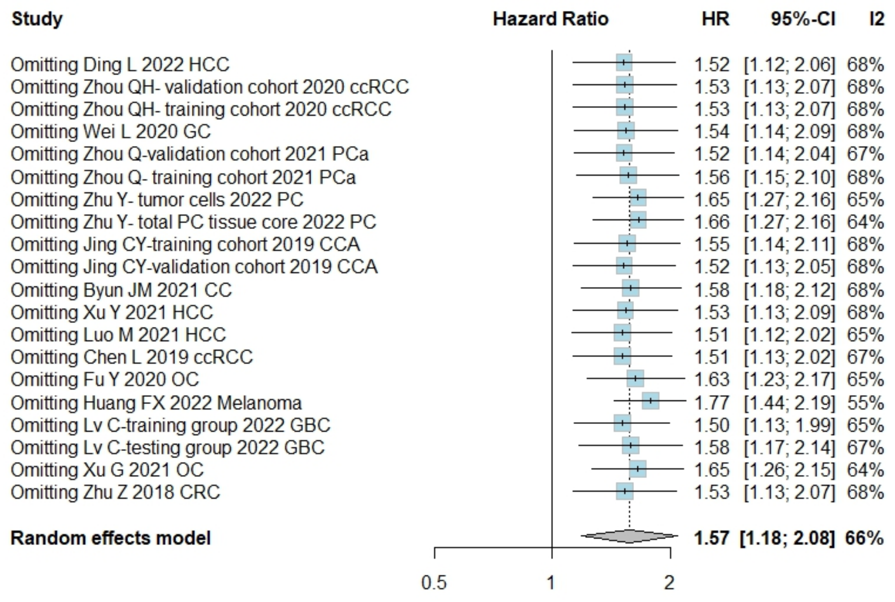

Figure S2C One-leave metaanalysis for investigating the effects of particular studies on the association between HHLA2 expression and overall survival in solid tumors. OS multivariate. High expression of HHLA2 was associated with poor OS (HR= 1,57, 95%CI: 1.18–2.08).

Figure S3A

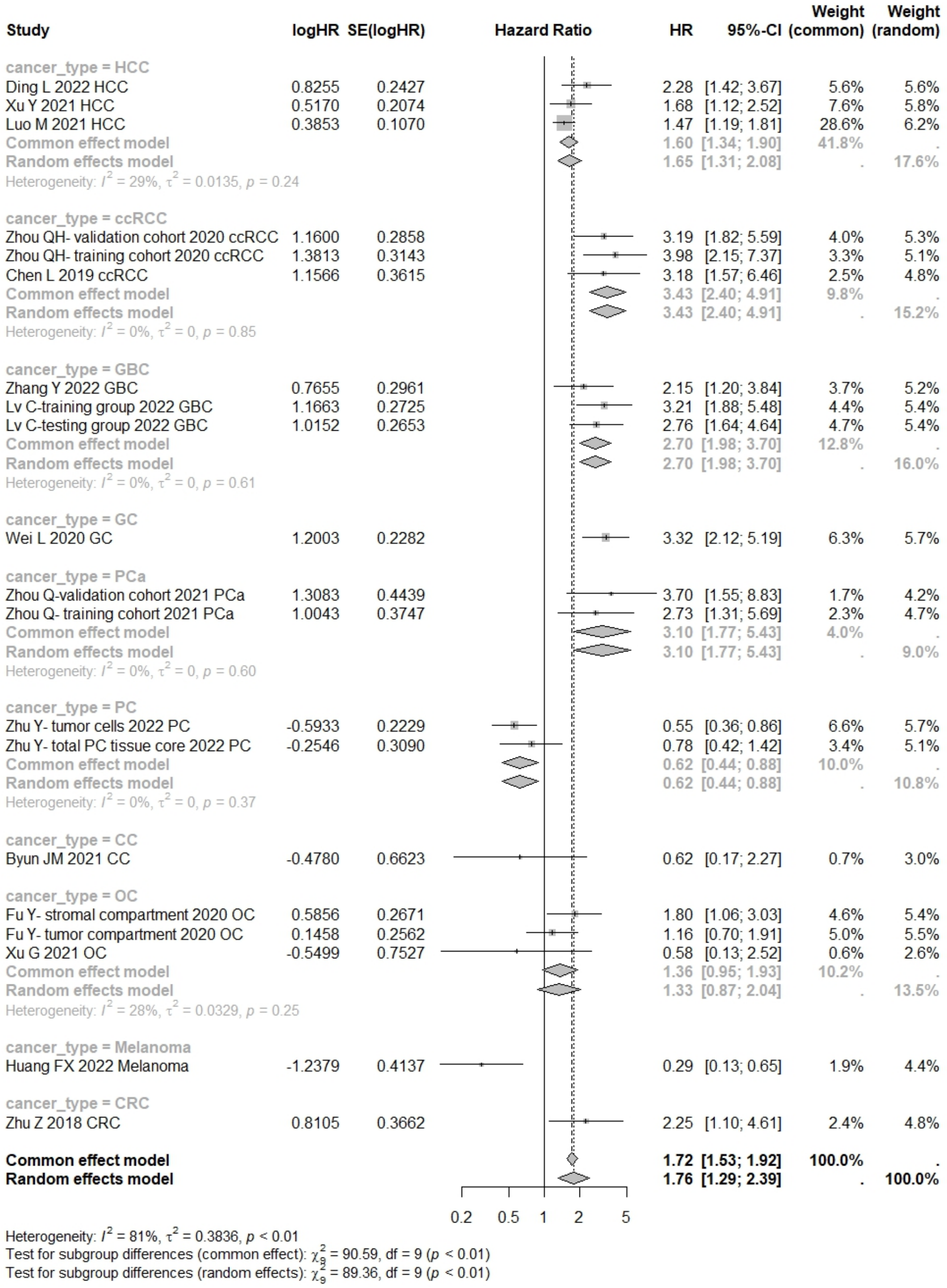

Figure S3A Subgroup analysis for the relationship between HHLA2 and overall survival. Grouped by different cancers. Univariate analysis. High expression of HHLA2 was associated with poor OS in hepatocellular carcinoma (HR = 1.60, 95% CI: 1.34-1.90), clear cell renal cell carcinoma (HR = 3,43, 95% CI: 2.40-4.91), gallbladder cancer (HR = 2.70, 95% CI: 1.98–3.70), prostate carcinoma (HR = 3.10, 95% CI: 1.77–5.43), apart from pancreatic cancer (HR = 0.62, 95% CI: 0.44-0.88). There was no significant relation between exression of HHLA2 and OS in ovarian cancer.

Figure S3B

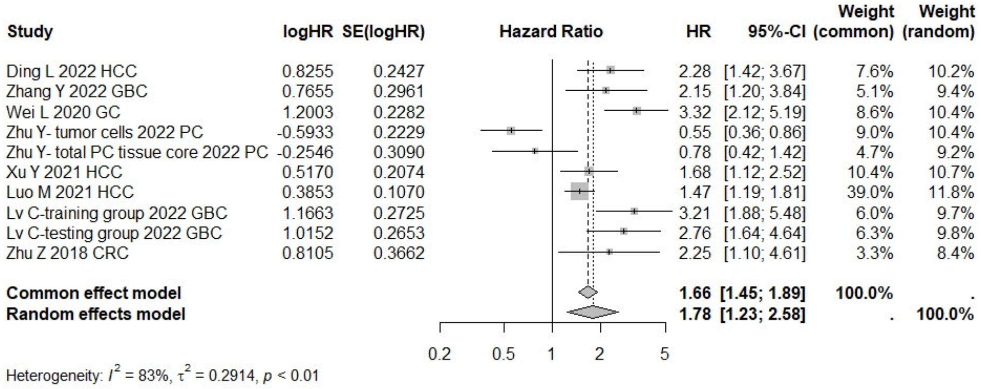

Figure S3B Forest plot of studies evaluating HRs of high HHLA2 expression and OS in gastrointestinal cancers. Univariate analysis. High expression of HHLA2 was associated with shorter OS (HR=1.78 95% CI: 1.23-2.58).

Figure S3C

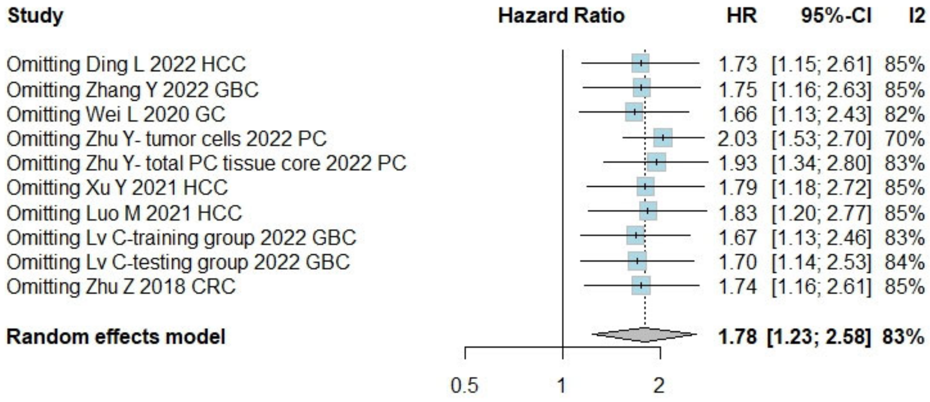

Figure S3C One-leave meta-analysis for investigating the effects of excluding particular studies from analysis on the relationship between high HHLA2 expression and OS in gastrointestinal cancers. High expression of HHLA2 was associated with poor OS (HR= 1 78, 95%CI: 1.23–2.58). Univariate analysis.

Figure S4A

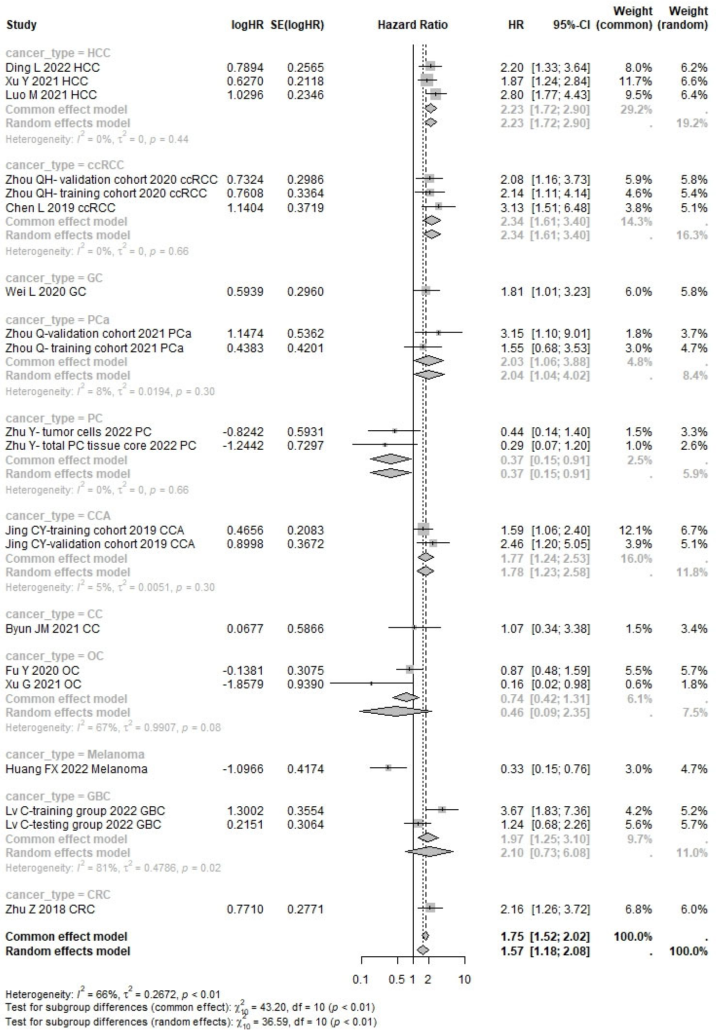

Figure S4A Subgroup analysis for the relationship between HHLA2 and overall survival. Grouped by different cancers. Multivariate analysis. High expression of HHLA2 was associated with poor OS in hepatocellular carcinoma (HR = 2.23, 95% CI: 1.72-2.90), clear cell renal cell carcinoma (HR = 2.34, 95% CI: 1.61-3.40), prostate carcinoma (HR = 2.03, 95% CI: 1.06–3.88), cholangiocarcinoma (HR = 1.77, 95% CI: 1.24-2.53), apart from pancreatic cancer (HR = 0.37, 95% CI: 0.15-0.91). There was no significant relation between exression of HHLA2 and OS in ovarian cancer and gallbladder cancer.

Figure S4B

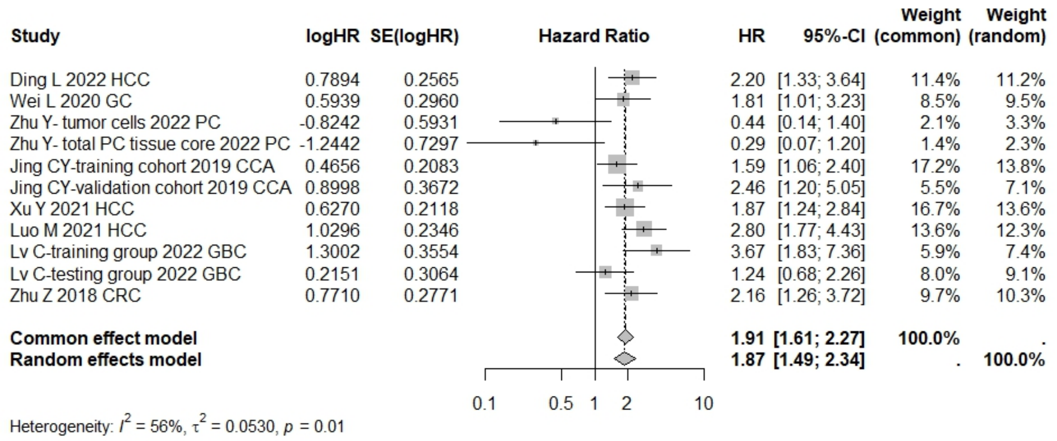

Figure S4B Forest plot of studies evaluating HRs of high HHLA2 expression and OS in gastrointestinal cancers.Multivariate analysis. High expression of HHLA2 was associated with poor OS (HR = 1.87, 95% CI: 1.49-2.34)

Figure S4C

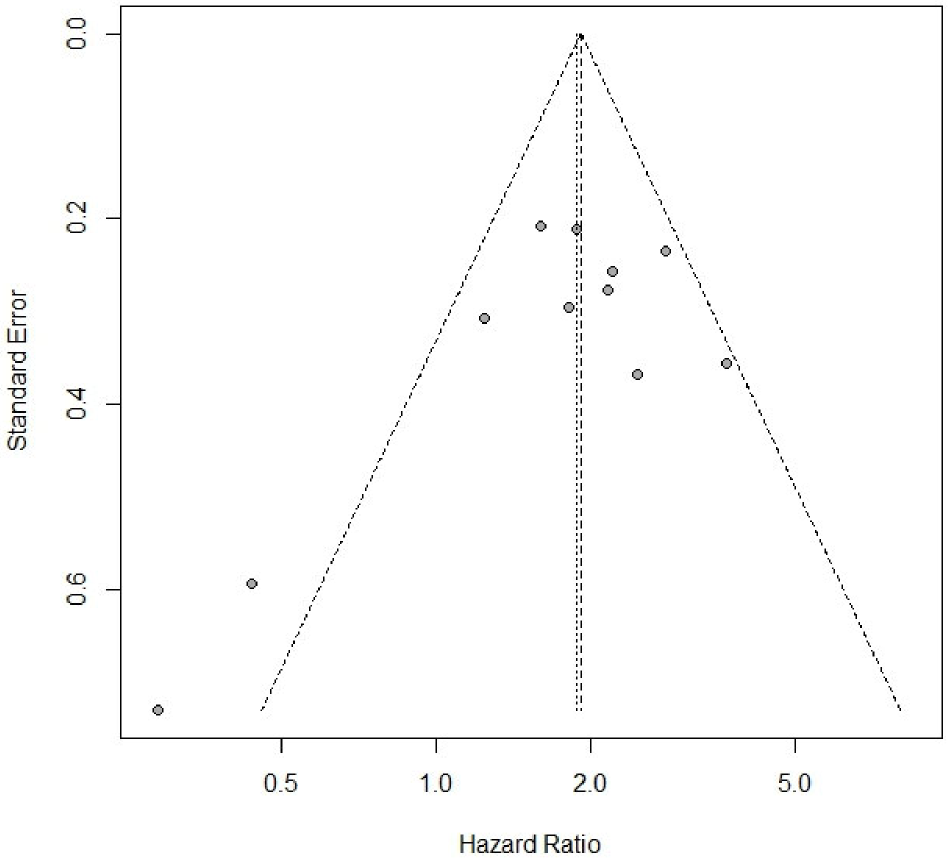

Figure S4C Funnel plots for evaluating potential publication bias on the association between HHLA2 high expression and overall survival in gastrointestinal cancers. Multivariate analysis.

Figure S5A

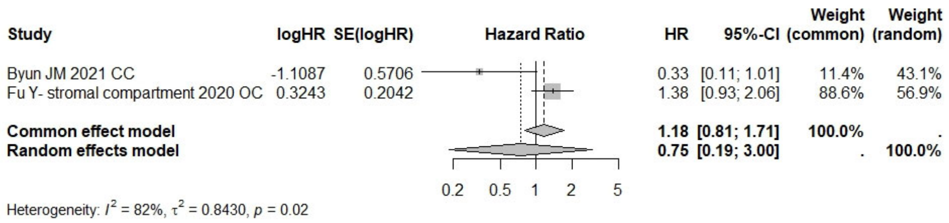

Figure S5A Forest plot for DFS. Univariate analysis. There was no association between HHLA2 expression and DFS.

Figure S5B

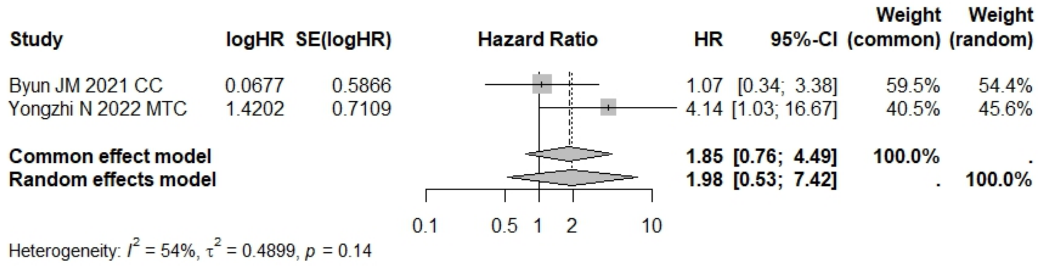

Figure S5B Forest plot for DFS. Multivariate analysis. There was no association between HHLA2 expression and DFS.

Figure S5C

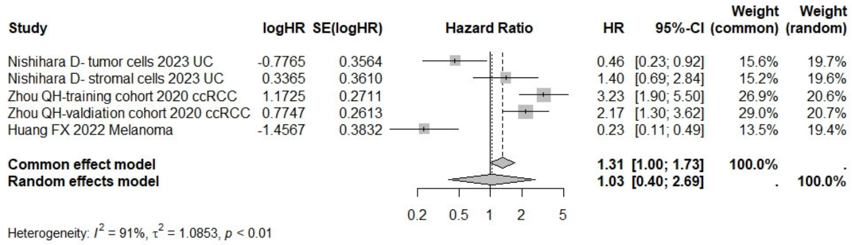

Figure S5C Forest plot for PFS. Univariate analysis. There was no association between HHLA2 expression and PFS.

Figure S5D

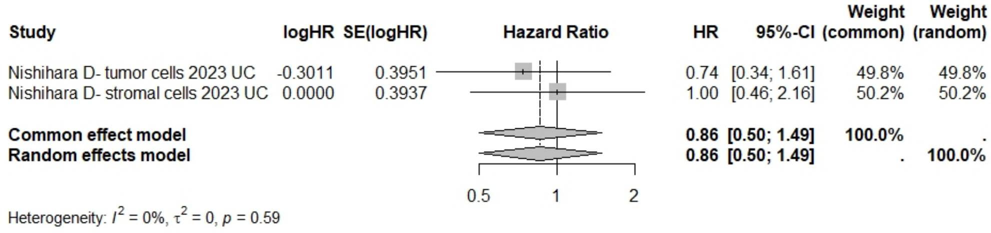

Figure S5D Forest plot for PFS. Multivariate analysis. There was no association between HHLA2 expression and PFS.

Figure S5E

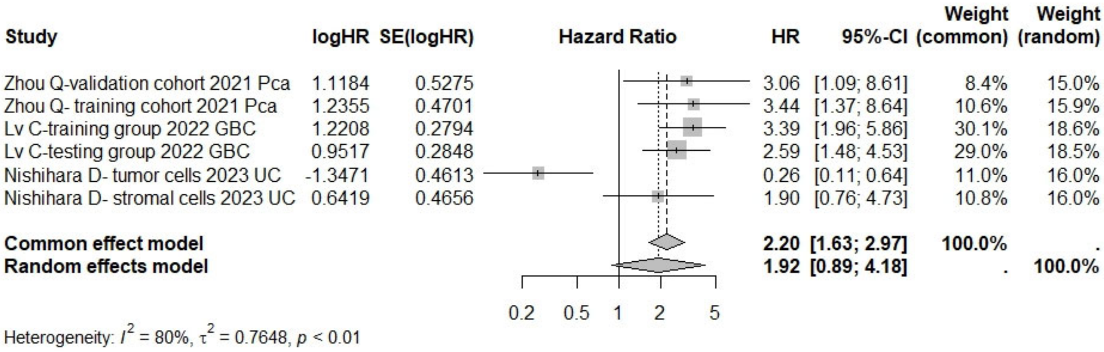

Figure S5E Forest plot for DSS. Univariate analysis. There was no significance between the high expression of HHLA2 and DSS.

Figure S5F

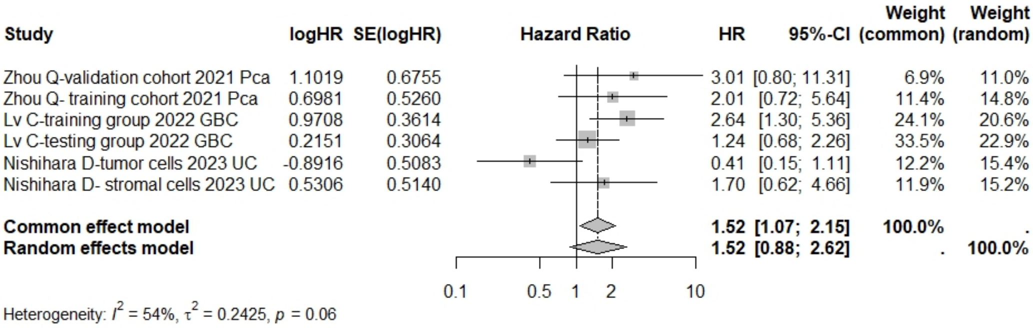

Figure S5F Forest plot for DSS. Multivariate analysis. There was no significance between the high expression of HHLA2 and DSS.

Figure S6

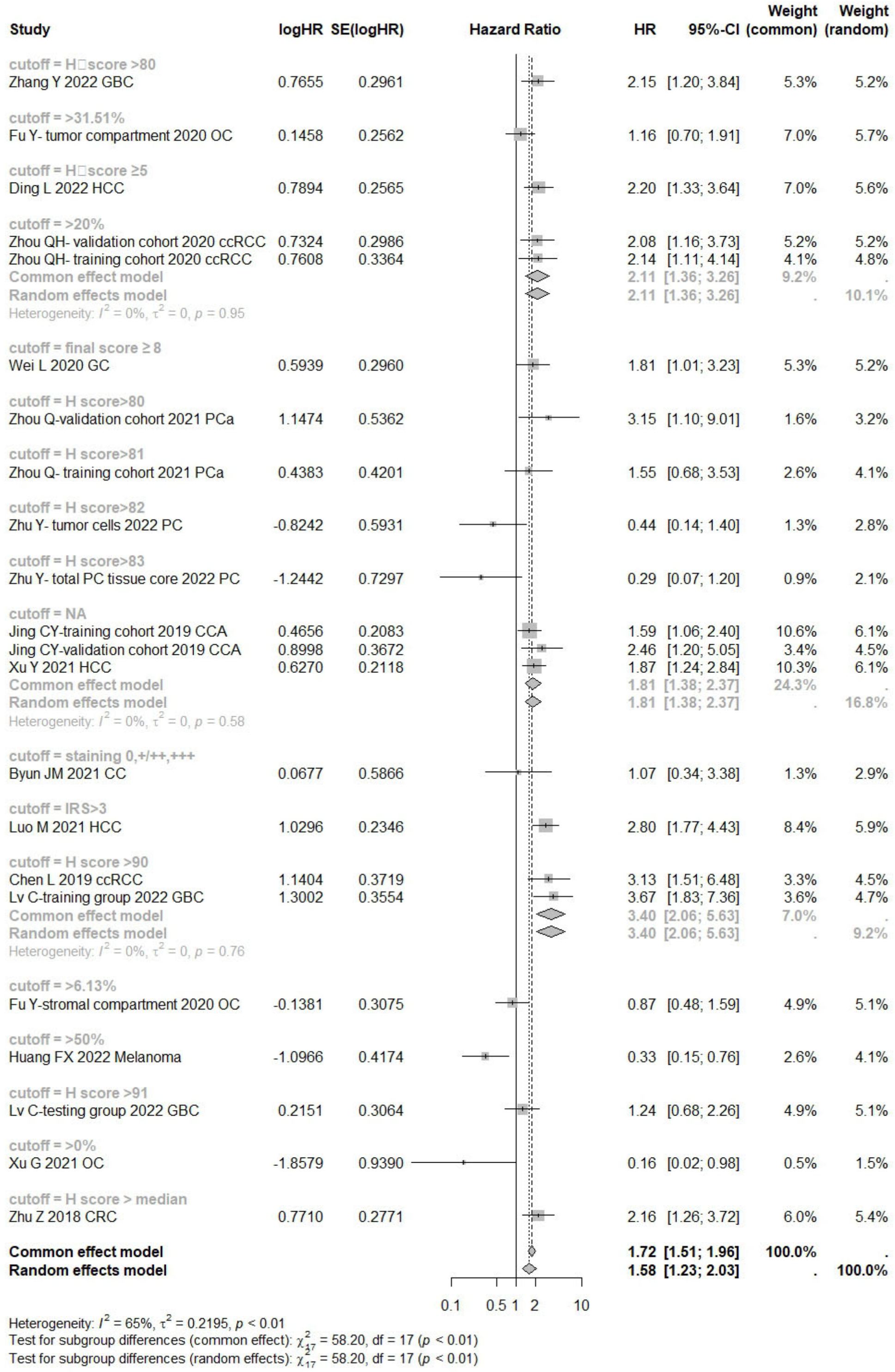

Figure S6 Subgroup analysis for the asssociation between HHLA2 and overall survival grouped by cut off of HHLA2 low/high expression
